# Effects of Whole-Body Electromyostimulation versus High-Intensity Resistance Exercise on Body Composition and Strength: A Randomized Controlled Study

**DOI:** 10.1155/2016/9236809

**Published:** 2016-02-29

**Authors:** Wolfgang Kemmler, Marc Teschler, Anja Weißenfels, Michael Bebenek, Michael Fröhlich, Matthias Kohl, Simon von Stengel

**Affiliations:** ^1^Institute of Medical Physics, Friedrich-Alexander University Erlangen-Nürnberg, 91052 Erlangen, Germany; ^2^Department of Sports Science, University of Kaiserslautern, 67663 Kaiserslautern, Germany; ^3^Department of Medical and Life Sciences, University of Furtwangen, 78048 Schwenningen, Germany

## Abstract

High-intensity (resistance) exercise (HIT) and whole-body electromyostimulation (WB-EMS) are both approaches to realize time-efficient favorable changes of body composition and strength. The purpose of this study was to determine the effectiveness of WB-EMS compared with the gold standard reference HIT, for improving body composition and muscle strength in middle-aged men. Forty-eight healthy untrained men, 30–50 years old, were randomly allocated to either HIT (2 sessions/week) or a WB-EMS group (3 sessions/2 weeks) that exercised for 16 weeks. HIT was applied as “single-set-to-failure protocol,” while WB-EMS was conducted with intermittent stimulation (6 s WB-EMS, 4 s rest; 85 Hz, 350 ms) over 20 minutes. The main outcome parameters were lean body mass (LBM) as determined via dual-energy X-ray absorptiometry and maximum dynamic leg-extensor strength (isokinetic leg-press). LBM changes of both groups (HIT 1.25 ± 1.44% versus WB-EMS 0.93 ± 1.15%) were significant (*p* = .001); however, no significant group differences were detected (*p* = .395). Leg-extensor strength also increased in both groups (HIT 12.7 ± 14.7%, *p* = .002, versus WB-EMS 7.3 ± 10.3%, *p* = .012) with no significant (*p* = .215) between-group difference. Corresponding changes were also determined for body fat and back-extensor strength.* Conclusion*. In summary, WB-EMS can be considered as a time-efficient but pricy option to HIT-resistance exercise for people aiming at the improvement of general strength and body composition.

## 1. Introduction

Time constraints are frequently reported as the main hindrance for frequent exercise; thus, time-saving exercise protocols are attractive to people seeking to increase their performance, attractiveness, and health. With respect to resistance exercise, low volume, high-intensity training (HIT) protocols seem to be the most time-efficient method to improve muscle mass and strength, independent of the ongoing debate whether resistance exercise with higher volume may be more effective in general [1–5]. However, alternative training technologies tailored to commercial applications may dispute this position. This includes in particular whole-body electromyostimulation (WB-EMS), which is becoming increasingly popular in Europe. Unlike the well-known local EMS application, WB-EMS technology is able to stimulate all the main muscle groups with dedicated intensity simultaneously. HIT and WB-EMS are often regarded as being similarly time efficient and safe; however, the few studies comparing the effects of both methods on muscle mass and/or strength did not show consistent results [6–10]. Nevertheless, commercial suppliers advertise “outcome effects” of up to 18-fold higher compared with conventional resistance exercise training. This promise is, however, primarily based on the misinterpretation of very pronounced* creatine-kinase* (CK) peaks after (too) intense initial WB-EMS application [11, 12], whereas data that clearly confirm the superiority of WB-EMS with respect to relevant outcomes (i.e., lean body mass, strength) are still lacking.

To estimate the comparative relevance of WB-EMS for improving body composition and muscle strength, we compared WB-EMS with the comparably time-efficient gold standard reference “HIT.” In order to conclude this issue, we conducted a randomized controlled trial with healthy but untrained males, 30–50 years old, aiming to improve their physical fitness and body composition. Based on the results of previous HIT [13] and WB-EMS [9, 14, 15] studies, our primary hypothesis was that HIT exercise training was significantly more effective for improving muscle mass and maximum strength compared with WB-EMS.

## 2. Methods

**Figure 1:**
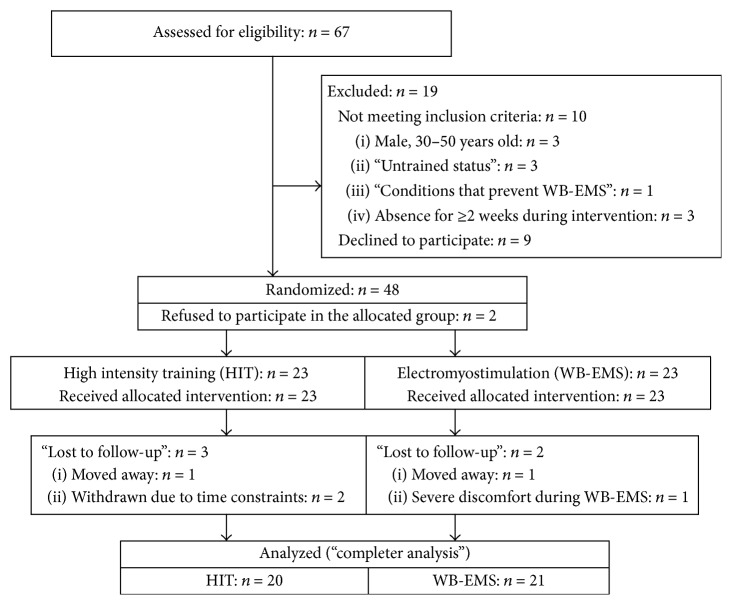
Flowchart of the study.

The aim of the study was to compare the effects of HIT-resistance training versus WB-electromyostimulation on body composition and strength in healthy but untrained middle-aged males living in the area of Herzogenaurach (Northern Bavaria, Germany). To adequately address our hypothesis, we conducted a 16-week single-blinded (in this section) randomized controlled exercise trial, using a parallel group design (Figure 1). The trial was planned and conducted by the Institute of Medical Physics, University of Erlangen (FAU), Germany. The study complied with the Declaration of Helsinki “Ethical Principles for Medical Research Involving Human Subjects” and was approved by the ethics committee of the FAU (Ethikantrag 245_13b) and the Federal Bureau of Radiation Protection (Z5-22462/2-2013-090). All the study participants gave written informed consent prior to study participation.

The study was registered under clinicaltrials.gov (NCT02078986). After the commencement of the trial, no further changes were made to the trial protocol. We adhered to the Consolidated Standards of Reporting Trial (CONSORT) for reporting (randomized) clinical trials [16].

### 2.1. Participants

Figure 1 gives the participant flow of the study. Using the public register, 1,500 male subjects between 30 and 50 years old living in the area of Herzogenaurach, Germany, were contacted in two blocks (September and November 2014). Personalized letters gave detailed study information including the most relevant eligibility criteria for the study. Sixty-seven males responded and were assessed for eligibility. Applying our inclusion criteria of (a) male, 30–50 years old; (b) “untrained status” defined as no regular resistance exercise training (<1 session/week) and less than an average of 90 min exercise/week at all; (c) lack of pathological changes of the muscle or heart or inflammatory diseases; (d) lack of medication/diseases affecting muscle metabolism; (e) conditions that prevent WB-EMS (e.g., epilepsy, cardiac pacemaker); and (f) absence of less than 2 weeks during the interventional period led to a total of 57 subjects being eligible. After informative meetings presenting the detailed study design, interventions, and measurements, nine subjects withdrew. The main reasons for withdrawal were unwillingness to join the randomization procedure (*n* = 5) and/or to conduct the WB-DXA assessment (*n* = 2). In order to increase compliance with the group allocation, the remaining 48 subjects were randomly allocated to one of the two study groups, (a) high-intensity training (HIT) group and (b) whole-body electromyostimulation (WB-EMS) group, by drawing lots. In detail, each of the 48 lots was placed in intransparent plastic shells (“Kinder Eggs,” Ferrero, Italy) and placed in a bowl so that participants and researchers never knew the allocation. Although subjects were requested to be free for both methods, two subjects allocated to the HIT-study arm immediately withdrew after randomization. In order to generate comparable baseline group sizes, however, the randomization sequence was correspondingly corrected by replacing a WB-EMS lot by a HIT lot. Thus, 23 HIT and 23 WB-EMS group subjects each embarked on the exercise program. All study participants were requested to maintain their physical activity and exercise habits during the study period.

Table 1 gives baseline characteristics of the participants. Randomization was effective; parameters that may have confounded our results did not vary significantly between the groups.

### 2.2. Procedures

Main outcome parameters are as follows:

- Total lean body mass (LBM) as assessed by whole-body dual-energy X-ray absorptiometry (WB-DXA).
- Maximum dynamic leg-extensor strength as assessed by an isokinetic leg-press device.

Secondary outcome parameters are as follows:

- Total body fat as assessed by WB-DXA.
- Maximum isometric back-extensors strength as assessed by an isometric test device.

### 2.3. Measurements

Each participant was tested at baseline and follow-up by the same researcher at the same time of the day (±1 hour). All follow-up tests were conducted after one week of rest (week 18). Tests were performed on one day within 60 min. Assessments were determined in a (semi)blinded mode. Accordingly, testing staff and outcome assessors were unaware of the participant status (i.e., WB-EMS or HIT) and were not allowed to ask.

#### 2.3.1. Anthropometry

Body height, weight, and waist circumference were measured by calibrated devices. Body Mass Index was calculated by weight (kg)/height (m^2^). Total and regional body composition was determined by dual-energy X-ray absorptiometry (QDR 4500a, Discovery Upgrade; Hologic, USA) using the default standard protocols of the manufacturer. Two researchers analyzed all the scans independently. Interrater reliability (intraclass correlation, ICC) for LBM was 0.92.

#### 2.3.2. Strength Parameters

Maximum strength of the leg extensors was determined using a ConTrex isokinetic leg-press (Physiomed, Laipersdorf, Germany). Bilateral concentric leg extension (and flexion) was performed in a sitting, slightly supine position (15°), supported by chest and hip straps. ROM was selected between 30° and 90° (knee angle), with the ankle flexed 90° and positioned on a flexible sliding footplate. The standard default setting of 0.5 m/s was used.

After warm-up and familiarization with the movement pattern, participants were asked to conduct five concentric repetitions (flexion/extension) with maximum voluntary effort. Participants conducted 2 maximum trials with two-minute rest in between; the higher value was used for data analysis. ICC for the maximum leg extension test is 0.88 (95% CI: 0.82–0.93) in our lab.

Maximum isometric strength of the back extensors was measured using a Schnell Isometric Tester (Schnell, Peutenhausen, Germany). Participants were positioned on the dynamometer seat in an upright position and were supported by thigh and hip straps. The participants had to press backwards (trunk extension) against the fixed lever arm touching the acromial site (extension). After two initial trials of low intensity, participants conducted 2 maximum efforts, each lasting 3–5 seconds, with a 40-second rest period in between. Again, the higher value was used for data analysis. For each measurement, the length and axis of the lever arm and the seat position of the participant were recorded to ensure optimum repeatability. Reproducibility of the isometric trunk strength tests (ICC) was 0.86 and 0.84 for back extension and flexion, respectively.

#### 2.3.3. Confounding Factors

A standardized questionnaire was applied to determine confounding factors that could affect the projected outcome parameters. Lifestyle, diseases and medications, and pain intensity and frequency at different skeletal sites were assessed at baseline and follow-up. Changes of physical activity and exercise were also determined by follow-up questionnaires [17] and personal interviews. ICC of the questionnaires were 0.78 [17] and 0.90. Individual dietary intake was assessed before and after trial by a 4-day protocol. The consumed food was analyzed using the Freiburger Ernährungsprotokoll (Freiburger Nutrition Protocol) (Nutri-Science, Hausach, Germany).

### 2.4. Study Procedure

Participants of the HIT and WB-EMS exercise group performed 16 weeks of either high-intensity exercise training or WB-EMS from November 2014 until March 2015 and from January 2015 until May 2015, respectively, in a well-equipped local gym. All the exercise sessions were consistently supervised; furthermore, participants recorded intensity, volume, and frequency of exercise in 4-week training logs. In both interventions (HIT and WB-EMS), all participants were requested to maintain their usual medication, dietary habits, physical activity, and exercise outside the trial protocol throughout the study course.

#### 2.4.1. Resistance Exercise Training (HIT) Protocol

In this study, HIT-resistance exercise was defined as single-set-to-failure protocol with intensifying strategies (manipulations of rest periods, time under tension, and exercise sequence load reduction). The exercise protocol scheduled two, rarely three (9th, 13th, and 16th week), consistently supervised exercise sessions per week on nonconsecutive days. All main muscle groups were addressed by 10–13 dedicated exercises/session, taken from a pool of 17 exercises (latissimus back and front pulleys, front chin-ups, seated rowing, back extension, inverse fly, hyperextension, sitting bench press, shoulder-press, military press, butterfly with extended arms, crunches, leg-press, leg extension, leg curls, and leg adduction and abduction) conducted on resistance devices (Technogym, Gambettola, Italy). While eight core exercises were applied in every session, the other exercises were prescribed in only one of the two or (rarely) three sessions/week.

During HIT period I, two weeks of initial conditioning with consistently 2 sets of 15 repetitions (reps.) and incomplete work to failure (maximum effort 2-3 reps.) was followed by two weeks of single sets with 8–10 repetitions with maximum effort (1 rep.). During this first 4-week period, movement velocity (time under tension: TUT) was consistently prescribed as the following: 2 s (concentric), 1 s (isometric), and 2 s (eccentric).

During the second 4-week period, the periodized HIT-training sequence started with the specification to work to momentary muscular failure (MMF). Prescribing maximum effort, the number of repetitions decreased linearly over 3 weeks (5th week, 8–10 reps., to 7th week, 3–5 reps.), with each 4th week planned as a “recreational week” with lower effort (maximum effort, 1 rep.). In detail, participants were requested to choose a load so that they could just perform the prescribed number of repetitions. Sets were always conducted to MMF, even when participants failed to realize the given number of repetitions. Rest periods were consistently set at 2 minutes between exercises. In parallel, movement velocity varied ranging from TUT “explosive” 1 s, 2 s for the higher repetition ranges (9-10 reps.) to 3 s, 1 s, and 4 s for the lower repetition ranges (3-4 reps.).

Additionally, during the third 4-week period, superset variations were introduced. Either agonist supersets (“compound sets”) using related muscle groups (i.e., back lat pulleys, seated rowing, and front chins) or antagonistic supersets (i.e., leg extension, leg curl, and leg-press) back to back with minor rest (<20 s) between the exercises and 2 minutes between the superset blocks were applied in alternating sessions. Using this concept, week 4 of this period was applied as a regeneration week with lower effort.

During the last 4 weeks (period IV) additional drop sets were introduced. In detail, after MMF, participants were requested to reduce the load and exercise again up to MMF. Single reductions of 10%–20% of the load were prescribed during the first two weeks; however, during the last two weeks, the load reduction of 10% was followed by another reduction of 5–20%; thus participants had to work 3 times to MMF. During the last period movement velocity was consistently prescribed as (TUT) 2 s, 1 s, and 2 s.

#### 2.4.2. Whole-Body Electromyostimulation (WB-EMS)

Because WB-EMS technology is a rather novel technology, a brief introduction will be given. Most innovative and different from the well-established local EMS, current WB-EMS equipment enables the simultaneous activation of up to 14–18 regions or 8–12 muscle groups (upper legs, upper arms, bottom, abdomen, chest, lower back, upper back, latissimus dorsi, and 4 free options) with different selectable intensities.

Adding up the stimulated area, 2,800 cm^2^ of body surface can be activated simultaneously. Strain or more precise current intensity can be individually selected and modified during the EMS session. The WB-EMS protocol applied in the present study scheduled the intermitted low intensity/low amplitude movement protocol slightly adapted from usual commercial settings and elaborately described in recent studies [14, 15, 18, 19]. In detail, participants conducted a consistently guided and supervised 20-minute WB-EMS session 3 times in 2 weeks (i.e., 1.5 times per week; each Monday or Tuesday and each second Thursday, Friday, or Saturday), always on two nonconsecutive days over 16 weeks. Groups of three participants were coached by a certified instructor; the session was also acoustically and visually guided by videos that exactly mimic the 6 s movement and 4 s rest rhythm of the protocol (see below). Using WB-EMS devices from miha bodytec® (Gersthofen, Germany), bipolar electric current was applied with a frequency of 85 Hz and a pulse breadth of 350 *μ*s intermittently with 6 s of EMS simulation to perform the movement and 4 s of rest (Table 2). Generally, the WB-EMS protocol closely followed the typical setting of commercial WB-EMS sessions with their low loading/low amplitude movement strategy. In summary, the 6 basic movements (“core exercises”) given in Table 2 were combined and slightly modified (e.g., twisted crunch) to generate 12 dynamic exercises that were performed without any additional weights in a standing position. Exercises were structured in 1-2 sets of 6–8 repetitions.

Amplitude, velocity, and corresponding intensity generated by the movement were set low (i.e., squat: leg-flexion: <35°) to prevent effects from the exercise per se. Additionally, no progressive increment of intensity with respect to the exercises was applied during the study phase. After a conditioning period of 5 WB-EMS sessions, current intensity was individually adapted in accordance with the participants in order to generate a rate of perceived exertion (RPE) of “hard” to “very hard” (Borg CR-10 Scale “6” of “10” (impossible) [20]). The corresponding current intensity was saved for each region on chip cards to generate a fast, reliable, and valid setting during the subsequent WB-EMS sessions. After this initial setting and a current conditioning period of 3–5 minutes, instructors slightly increased the current intensity every 3–5 minutes in close cooperation with the participants to maintain the RPE of “hard” to “very hard” during the session.

### 2.5. Statistical Analysis

The a priori sample size calculation referred to lean body mass. Based on a sample size of 21 subjects per group and a Type 1 Error of 5%, the statistical power (1 − *β*) to detect a 10 ± 10% difference between the groups was 90%. Assuming a dropout rate of 20%, our goal was to recruit 25 participants per group.

The data were analyzed following a finisher analysis; for example, all the participants who took part in the follow-up measurements were included in the analysis irrespective of their compliance. Baseline and follow-up data are reported as mean values and standard deviations.

Changes between baseline and follow-up in HIT and WB-EMS were reported both as absolute (tables) and as percentage changes (text). In addition, mean differences (with 95% confidence intervals) between HIT and WB-EMS based on absolute changes were reported in Table 3. Differences of baseline characteristics (Table 2) were checked by Welch *t*-test. Where applicable (normal data distribution), analyses of variance with repeated measurements adjusted for baseline values were performed to check time × group interactions; otherwise, Welch *t*-test based on absolute differences was used. All tests were 2-tailed, and statistical significance was accepted at *p* < .05. Effect sizes (ES) were calculated using Cohen's *d*′. SPSS 21.0 (SPSS Inc., Chicago, IL) was used for all statistical procedures.

## 3. Results

During the interventional period of 16 weeks, 3 participants of the HIT and 2 participants of the WB-EMS group were lost to follow-up. As described above, two subjects refused to join their allocated intervention (HIT) and quit the study immediately after randomization. Reasons for withdrawal were (a) job related relocation (HIT: *n* = 1; WB-EMS: *n* = 1), (b) job related time constraints (HIT: *n* = 2), and (c) severe discomfort during the WB-EMS application (*n* = 1).

Relative attendance rate was comparable between the groups (HIT 93.3 ± 7.0% versus WB-EMS 89.5 ± 10.7%; *p* = .171); net length of training sequence (exercise protocol only), however, varied significantly (*p* < .001) between the groups (HIT 30.3 ± 2.3 versus WB-EMS 20 ± 0 minutes). However, the differences for total “time under load” between HIT and WB-EMS (WB-EMS 242 ± 22 versus HIT 365 ± 46 min, *p* < .001) did not fully reflect the difference in total training volume (WB-EMS 403 ± 37 versus HIT 847 ± 87 min, *p* < .001).

As stated, perceived exercise intensity of the WB-EMS participants was consistently adjusted to an RPE of 6 (5 = “hard,” 7 = “very hard”) during the session. In parallel, the HIT participants' regular training logs demonstrated a corresponding RPE of 4.75 ± .28 for the first 4-week period, 5.64 ± 4.4 for the second period, 6.42 ± .39 for the third period, and 7.31 ± .36 for the last 4-week period, without considering the “recreational weeks.”

During the study course, no relevant negative side effects with respect to musculoskeletal lesions or diseases related potentially to the study intervention were recorded.

### 3.1. Main Outcome Parameters

Table 3 lists baseline, follow-up, and corresponding changes and group differences for LBM and maximum leg-extensor strength. At baseline, borderline significant differences were observed for maximum leg-extensor strength but not for LBM. However, analysis was consistently adjusted to baseline values.

LBM increased significantly (*p* = .001) in both groups (HIT 1.25 ± 1.44% versus WB-EMS 0.93 ± 1.15%) with no significant differences between the two groups (*p* = .395). In parallel, the significant changes (*p* < .001) of appendicular skeletal muscle mass (i.e., lean soft tissue of the upper and lower limbs; not given in Table 3) in the WB-EMS and HIT group (0.48 ± 0.41 versus 0.60 ± 0.45 kg, *p* = .341) confirmed the results of the LBM assessment.

With respect to changes of regional LBM, we observed a slight trend to more favorable trunk-LBM changes in the HIT group (*p* = .635), similar changes for the lower limbs (*p* = .968), and 63% higher upper limb LBM changes in the HIT group (*p* = .039), indicating that LBM changes were not uniform.

Maximum leg-extensor strength changed favorably in both groups (HIT 12.7 ± 14.7%, *p* = .002, versus WB-EMS 7.3 ± 10.3%, *p* = .012) with nonsignificant (*p* = .215) higher changes among the HIT group. Isometric back extension strength increased significantly (*p* < .001) in both groups (HIT 10.2 ± 8.8% versus 11.6 ± 10.0%) with no significant group difference (*p* = .663).

Total body fat mass decreased significantly in both groups (HIT −4.4 ± 7.5%, *p* = .035, versus WB-EMS −3.7 ± 3.9, *p* = .001). Differences with respect to body fat changes adjusted for baseline total body fat mass were nonsignificant (*p* = .829).

Thus, we have to reject our hypothesis that HIT-resistance training was significantly more effective for improving muscle mass and maximum strength than WB-EMS.

### 3.2. Secondary Outcome Parameters

Secondary outcome parameters were given in Table 4.

### 3.3. Confounding Parameters

With respect to relevant diseases, 5 participants listed treated hypertension (HIT: *n* = 2), 5 reported slight allergic respiratory disorders (HIT: *n* = 1), 2 suffered from depression (HIT: *n* = 1), and 3 men stated resection of the thyroid or hypothyroidism (HIT: *n* = 1). No relevant changes of disease status were reported after the interventional period. As per the study criteria, participants receiving medication affecting the musculoskeletal system were not included. Further, apart from discontinued hypertension treatment in two participants, no relevant changes of medication during the study period were reported.

Changes of occupational and leisure time physical activity (*p* ≥ .650) were slight and did not differ between the groups (*p* = .793). Further, average exercise participation and weekly exercise volume did not change significantly in the HIT or WB-EMS. However, in response to specific inquiries, two participants (HIT, *n* = 1, versus WB-EMS, *n* = 1) admitted having performed endurance exercise training (running) with an average volume of 2 and 2.5 hours/week in order to reduce body fat (Table 4).

Energy uptake increased nonsignificantly in the HIT (2.9 ± 9.9%, *p* = .413) and significantly in the WB-EMS group (7.8 ± 10.6%, *p* = .010); however, group differences were not significant (*p* = .159). In parallel, relative protein intake (g/kg/d) increased in both groups (HIT 8.3 ± 21.6%, *p* = .349, versus WB-EMS 11.0 ± 17.5%, *p* = .030) with no significant differences between the groups (*p* = .685). Of importance, no participants said that they had reduced energy uptake in order to reduce weight or body fat.

## 4. Discussion

Time-efficient exercise protocols may be the best choice for improving fitness and body composition of subjects with limited time resources. In the area of resistance exercise, two methods, namely, high-intensity training (HIT) and whole-body electromyostimulation (WB-EMS), were identified as candidates that satisfy the time-effectiveness requirement. In respect to body composition, only a few studies determined the effect of WB-EMS on body fat and/or fat-free mass in healthy young or middle-aged cohorts [6–10]. Two of the three studies that addressed lean body mass reported significant increases of total LBM ([10]: % not given, [9]: 1.9%) along with significant reductions of body fat mass (5% and 7%, resp.). In contrast, Boeckh-Behrens et al. [6–8] listed either no effects [6, 7] or significant fat gains [8] in their cohort of sports students albeit with (very) low body fat using a suboptimum test device. The favorable effect of WB-EMS on muscle mass parameters (e.g., cross-sectional area (CSA), fiber size, and girth) was confirmed by studies that conducted local EMS application in healthy nonathletic, nonparalyzed subjects [21–25]. While no comparative studies were available for WB-EMS, the few studies that compared the effect of local EMS and volitional contraction on muscle mass in healthy nonathletic persons determined comparable significantly positive (CSA) changes through both methods [21, 25]. However, although we generally confirmed these results, our approach was much more pragmatic and focused on comparing two time-efficient training methods with respect to endpoints (e.g., body composition) relevant for the potential user.

With respect to strength gains, the significant positive effect of WB-EMS in healthy, untrained subjects is undisputed [26, 27]. The maximum isometric and/or dynamic strength gain of the present study is comparable to data given for WB-EMS application in studies with trained cohorts (*n* = 5) [6–8, 14, 28]. Interestingly, studies that applied local EMS reported higher average isometric (up to 58%) or dynamic maximum (up to 80%) strength gains with more favorable results in trained or elite athletes compared with untrained subjects [27].

More relevant for this topic is the question of whether EMS-induced strength gains were similar to traditional resistance exercise training in untrained healthy cohorts with higher training volume. Unfortunately, different protocols for resistance exercise and EMS along with varying endpoints and muscle areas addressed prevent a clear decision. A simple comparison of EMS applications and resistance training with respect to strength parameters (i.e., power, maximum strength) without considering any further specification showed either superiority of EMS [29], of volitional resistance exercise training [30, 31], or no difference [25, 32, 33], at least in untrained healthy subjects. Hainaut and Duchateau [34] conclude after an early review of the literature that there is broad agreement “that the force increases induced by EMS (NMS) are similar to, but not greater than, those induced by voluntary training.” However, it should be considered that the levels of evidence generated by these studies conducted in the eighties are only moderate.

Some study features and limitations may reduce the impact of our results: (1) compared with other studies [35] focusing on LBM in adults, the study was relatively short (16 weeks); further, we did not apply intermitted tests. Thus, (a) we cannot exclude the possibility that we did not assess the main effect of the exercise protocols on LBM and (b) were unable to evaluate strength kinetics. (2) We failed slightly to reach our calculated sample size of 25 participants/group; however, the dropout rate was lower than expected. Hence, the power of the study ought to be sufficient to detect relevant effects. (3) We did not adjust either protocol for exercise parameters (e.g., exercise volume). Instead, we focused on a real-world comparison of a novel exercise technology versus a “gold standard” reference protocol with the common denominator (low) time expenditure. However, with respect to exercise intensity, we tried to apply comparable prescriptions of exercise intensity via RPE. (4) The exercise protocol of the HIT group was very strenuous; however, due to the low training frequency and regular regeneration periods, we did not expect that results were confounded by overreaching symptoms. (5) The assessment of exercise intensity by RPE (Borg CR-10 Scale) may be critical because this tool has so far been validated by voluntary exercise. However, we think it is legitimate to use RPE in this context at least under the premise that other more objective approaches to identify and prescribe exercise intensity during WB-EMS and HIT are not available/applicable. (6) We focused on untrained middle-aged men assuming that both WB-EMS and HIT-resistance exercise training may be equally attractive and feasible for this cohort and hence this topic may be of high interest with respect to health promotion. Further, a comparison of EMS and resistance exercise in trained or athletic cohorts may be defective due to previous adaption to voluntary exercise in these cohorts.

## 5. Conclusion

In summary, we observed comparable or at least similar increases of muscle parameters after 16 weeks of WB-EMS compared with the reference method “HIT.” Thus, WB-EMS can be considered as an attractive, time-efficient, and effective option to HIT-resistance exercise for people seeking to improve general strength and body composition. On the other hand, due to the close supervision of present WB-EMS applications, this exercise technology is much more expensive. However, taking into account the fact that WB-EMS technology will become more feasible and cost efficient over the next few years, the application of WB-EMS will be increasingly implemented in commercial and noncommercial fitness settings.

## Figures and Tables

**Table 1:** Baseline characteristics of the participants of the HIT and WB-EMS group.

Variable	HIT *n* = 23	WB-EMS *n* = 23	Difference (*p*)
Age [years]^a^	41.9 ± 6.4	43.7 ± 6.1	.429
Body height [cm]	181.6 ± 5.6	179.3 ± 6.3	.197
Body weight [kg]	88.8 ± 12.5	91.5 ± 12.8	.471
BMI [kg/m^2^]	26.9 ± 3.3	28.5 ± 4.1	.151
Total body fat DXA [%]	24.7 ± 4.8	26.5 ± 5.2	.220
Physical activity [index]^a^	2.91 ± 1.08	3.22 ± 1.51	.463
Exercise volume [min/week]	45.9 ± 37.8	50.2 ± 35.2	.689
Energy intake [kcal/d]^b^	2346 ± 463	2387 ± 712	.828
Protein intake [g/kg/d]^b^	1.07 ± 0.27	1.10 ± 0.28	.695
Alcohol [g/d]^b^	10.0 ± 9.4	12.1 ± 10.0	.514
Smoker [*n*]	7	6	.743

^a^Self-rated physical activity score (1 to 7, 1: very low; 7: very high) [17]; ^b^assessed by a 4-day dietary protocol and analyzed using the “Freiburger Ernährungsprotokoll” (Freiburger Nutrition Protocol, Nutri-Science, Germany).

**Table 2:** “Core exercises” applied during WB-EMS.

Exercise movements	
(1) Squat (6 s down) and vertical chest press/squat (6 s up) and vertical rowing	
(2) Squat (6 s down) and lat pulldown/squat (6 s up) with military press	
(3) Deadlift (6 s down) with arm-curls (ext.)/deadlift (6 s up) with arm-curls (flex.)	
(4) Squat (6 s down), crunch with butterfly/squat (6 s up) and reverse fly	
(5) Squat (6 s down) and trunk flexion (crunches); return to upright position	

**Table 3:** Baseline and follow-up data, absolute changes, and statistical parameters of primary endpoints in the HIT, WB-EMS, and control group.

	HIT (*n* = 20) (MV ± SD)	WB-EMS (*n* = 22) (MV ± SD)	Difference MV (95% CI)	*p*	Effect size (*d*′)
Lean body mass [kg]^a^					
Baseline	68.24 ± 7.38	67.49 ± 7.33	—	.875	—
16 weeks	69.10 ± 7.23	68.12 ± 7.42	—	—	—
Difference	.855 ± .973 (.001)	.625 ± .775 (.001)	.230 (−.324 to 785)	.395	0.26

Maximum leg extension strength (leg-press) [N]					
Baseline	3201 ± 783	3605 ± 506	—	.050	—
16 weeks	3608 ± 467	3869 ± 218	—	—	—
Difference	408 ± 521 (.002)	264 ± 448 (.012)	144 (−.159 to 447)	.215	0.30

^a^ *n* = 21 in the WB-EMS group.

**Table 4:** Baseline and follow-up data, absolute changes, and statistical parameters of secondary endpoints in the HIT, WB-EMS, and control group.

	HIT (*n* = 20) (MV ± SD)	WB-EMS (*n* = 22) (MV ± SD)	Difference MV (95% CI)	*p*	Effect size (*d*′)
Maximum isometric back extension strength [N]					
Baseline	289.9 ± 73.1	291.5 ± 62.7	—	.939	—
16 weeks	319.4 ± 70.4	325.3 ± 69.3	—	—	—
Difference	29.5 ± 19.8 (<.001)	33.8 ± 28.4 (<.001)	3.3 (−12.2 to 18.8)	.663	.18

Total body fat [kg]^a^					
Baseline	23.09 ± 7.00	24.32 ± 7.23	—	.259	—
16 weeks	22.07 ± 6.78	23.41 ± 7.00	—	—	—
Difference	1.02 ± 2.01 (.035)	.91 ± 1.00 (.001)	.230 (−.324 to 785)	.829	0.07

^a^ *n* = 21 in the WB-EMS group.
